# A strategy of gene overexpression based on tandem repetitive promoters in *Escherichia coli*

**DOI:** 10.1186/1475-2859-11-19

**Published:** 2012-02-06

**Authors:** Mingji Li, Junshu Wang, Yanping Geng, Yikui Li, Qian Wang, Quanfeng Liang, Qingsheng Qi

**Affiliations:** 1 State Key Laboratory of Microbial Technology, Shandong University, Jinan 250100, Peoples Republic of China; 2 National Glycoengineering Research Center, Shandong University, Jinan 250100, Peoples Republic of China

**Keywords:** Promoter cluster, Tandem repeats, Gene overexpression, Metabolic engineering, Polyhydroxybutyrate

## Abstract

**Background:**

For metabolic engineering, many rate-limiting steps may exist in the pathways of accumulating the target metabolites. Increasing copy number of the desired genes in these pathways is a general method to solve the problem, for example, the employment of the multi-copy plasmid-based expression system. However, this method may bring genetic instability, structural instability and metabolic burden to the host, while integrating of the desired gene into the chromosome may cause inadequate transcription or expression. In this study, we developed a strategy for obtaining gene overexpression by engineering promoter clusters consisted of multiple core-*tac-*promoters (MCP*tac*s) in tandem.

**Results:**

Through a uniquely designed *in vitro *assembling process, a series of promoter clusters were constructed. The transcription strength of these promoter clusters showed a stepwise enhancement with the increase of tandem repeats number until it reached the critical value of five. Application of the MCP*tac*s promoter clusters in polyhydroxybutyrate (PHB) production proved that it was efficient. Integration of the *phaCAB *genes with the 5CP*tac*s promoter cluster resulted in an engineered *E.coli *that can accumulate 23.7% PHB of the cell dry weight in batch cultivation.

**Conclusions:**

The transcription strength of the MCP*tac*s promoter cluster can be greatly improved by increasing the tandem repeats number of the core-*tac*-promoter. By integrating the desired gene together with the MCP*tac*s promoter cluster into the chromosome of *E. coli*, we can achieve high and stale overexpression with only a small size. This strategy has an application potential in many fields and can be extended to other bacteria.

## Background

The balanced flux of the whole metabolic pathway *in vivo *is an important issue for accumulation of the desired metabolites [1]. However, many rate-limiting steps may exist in the pathways due to weak expression of the inherent gene(s) or lack of certain essential gene(s) [2-4]. Thus, homologous or heterologous overexpression of the desired genes at the rate-limiting steps is nearly an indispensable means during metabolic engineering [5]. As a useful and easy-to-manipulate tool, the plasmid-based expression system is generally engaged to achieve this purpose [6]. However, some disadvantages make it imperfect when plasmids are employed in the pathway engineering, such as genetic instability, structural instability and metabolic burden [7-9]. In addition, it can also become a intractable problem when the resulting plasmid is too large to be transformed into competent cells [10].

With the development of molecular biotechnology, many attempts have been made to overcome these flaws. Integration of the desired genes into the chromosome of the host seems able to circumvent these problems; and therefore many chromosome integration strategies have been developed in the past few years [11-15]. However, these approaches cannot obtain sufficient gene expression due to the inadequate strength of the promoter or the scant copy number of the target gene which was integrated into the chromosome. Repeated insertion of the target gene at multiple locations may improve its expression level to a certain degree through a site-specific chromosomal integration method developed by Kuhlman et al. [16]. Recently, Tyo et al. developed a plasmid-free method which can achieve high copies of the desired genes by chemically inducible chromosomal evolution method (CIChE) [17]. In this method, the strain of which the desired genes together with the antibiotics had been integrated into the chromosome was evolved to obtain high copies in the locus through gradually increasing antibiotic concentration in the medium. This strategy provided an available plasmid-free method to improve stable gene expression by increasing the copy number of interest genes in the chromosome, and therefore improved the efficiency of metabolic engineering. Yet each one-copy increase of the target gene in this method, it would bring an additional antibiotic resistance gene (*cat*) and a homologous region of 1 kb in the chromosome. Therefore, there would finally produce significant amounts of the superfluous DNA sequences when enough gene copies were achieved. These DNA sequences in the chromosome may also be metabolic burden for the host [18].

To maintain high expression of the desired genes but avoid superfluous DNA sequence, we developed a strategy to improve transcription strength by constructing the promoter clusters consisted of multiple core-*tac*-promoters (MCP*tac*s) in tandem. With an ingenious design, the series MCP*tac*s promoter clusters were assembled via the Gibson's method [19]. The transcription strength of the MCP*tac*s promoter clusters was then analyzed employing the green fluorescence protein (GFP) as an indicator. Application of the MCP*tac*s promoter clusters in polyhydroxybutyrate (PHB) production proved its efficiency and simplicity.

## Results

### Construction of the MCP*tac*s promoter clusters

The *tac *promoter is nearly the strongest available promoter for metabolic engineering. However, even the *tac *promoter cannot satisfy the need of high expression of the desired genes in the pathway engineering, especially when they should be integrated into the chromosome. Therefore, to improve the expression of the desired genes in the chromosome, we came up with the idea of constructing a series promoter clusters by tandem repeating the strong *tac *promoter. Our preliminary experiments showed that the core-*tac*-promoter (containing 41 bp) possessed complete transcription function and almost the same transcription strength as the wild type *tac *promoter. To minimize the size of the promoter clusters for easy-to-construct, only the constitutive core-*tac*-promoter was then employed.

**Figure 1:**
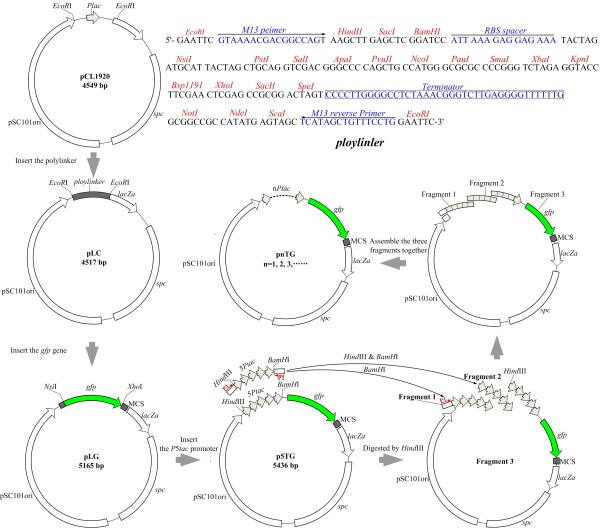
**Construction outline of the MCP*tac*s promoter clusters**. Fragment 5CP*tac*s with the flanking sequence was amplified by PCR with p5TG as the template. Fragment 1 was generated by digesting fragment 5CP*tac*s with *BamH*I. Fragment 2 was digested from fragment 5CP*tac*s with *BamH*I and *Hind*III. Fragment 3 was linearized from the plasmid p5TG with *Hind*III. Then, the three fragments were assembled together under the action of T5 exonuclease, Phusion DNA polymerase and Taq DNA ligase in the isothermal process.

For construction, we chose the low-copy plasmid pCL1920 as the cloning vector and the *gfp *gene (Green fluorescence protein, GFP) as the transcription strength indicator. The series constitutive MCP*tac*s promoter clusters were constructed *in vitro *following the Gibson's assembling method (See method section) [19]. The key point in our assembling process was that all the three assemble fragments had the same designed repetitive overlapping sequence, which consisted of five core-*tac*-promoters aligned in tandem (The 5CP*tac*s promoter cluster) (Figure 1). In the assemble process, every core-*tac-*promoter could overlap with anyone in the additional assembling fragments or the same fragment (for fragment 2). Meanwhile, the 5CP*tac*s promoter cluster can be cut into any number, from one to four, of the core*-tac-*promoter under the action of T5 exonuclease and a high fidelity Phusion Host Start DNA Polymerase. Thus, plasmids with arbitrary positive integer of the core-*tac-*promoter were assembled [19]. As a result, ten plasmids with sequentially increased number, beginning from one, of the repetitive core*-tac*-promoter were obtained, and were named p1TG, p2TG, p3TG, p4TG, p5TG, p6TG, p7TG, p8TG, p9TG and p10TG, respectively.

### Characterization of the MCP*tac*s promoter clusters

**Figure 2:**
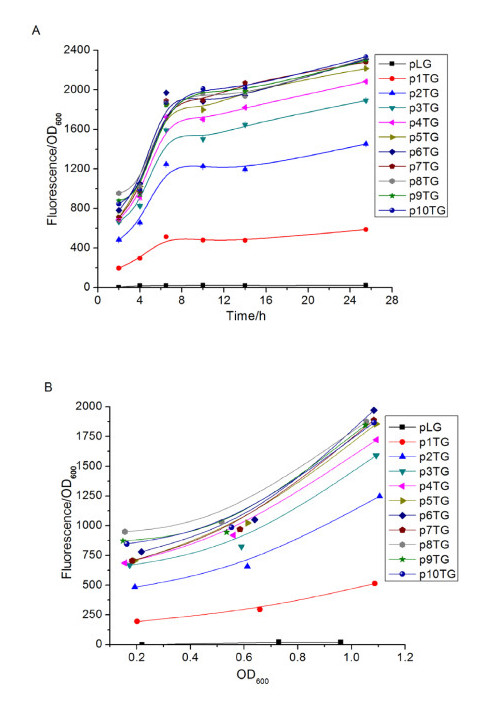
**Determination of the transcription strength of the MCP*tac*s promoter clusters by fluorescence analysis**. (A) The time dependent analysis of the fluorescence/OD_600 _ratio; (B) The OD_600 _dependent analysis of the fluorescence/OD_600 _ratio.

**Figure 3:**
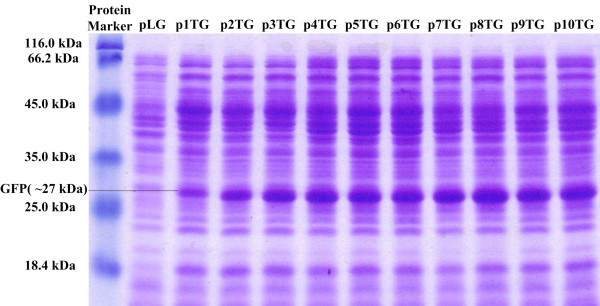
**Determination of the GFP expression of the MCP*tac*s promoter clusters by SDS-PAGE**. After 16 h cultivation, cells were harvested and lysed completely in isometric PBS. 15 μL supernatant containing GFP was subjected to SDS-PAGE. GFP bands were measured with ImageJ software, and the areas of their corresponding peaks were used to denote the relative GFP amounts. Lane*1 *Protein Marker, lane*2 *5874.43, lane*3 *12432.74, lane*4 *14813.91, lane*5 *17585.91, lane*6 *18039.91, lane*7 *18487.33, lane*8 *17334.79, lane*9 *16975.38, lane*10 *20819.62, lane*11 *18758.20, lane*12 *20396.74.

To characterize the constructed MCP*tac*s promoter clusters, fluorescence intensity of each construct radiated from the green fluorescence protein (GFP) was determined (Figure 2). *E.coli *DH5***α ***harboring these plasmids were cultivated in LB medium. By analyzing the cell growth and relevant fluorescence of each strain, we found that the more copies of the tandem repetitive core*-tac-*promoter, the higher value of fluorescence/OD_600 _(Figure 2A). This suggested that the transcription strength can be enhanced by increasing the tandem repeats of the core*-tac-*promoter. The fluorescence reached almost the maximum if the tandem repetitive number of the core*-tac-*promoter in the construct was five (The 5CP*tac*s promoter cluster). The fluorescence intensity of the 5CP*tac*s promoter cluster was about 4.4-fold more than that of the original core*-tac-*promoter. The value of fluorescence/OD_600 _to OD_600 _taken from the logarithmic phase was also analyzed to exclude effect of the cell growth on fluorescence enhancement. It also exhibited a stepwise enhancement for the same OD_600 _with the increase of tandem repeats. This result proved that the fluorescence/OD_600 _enhancement was due to the increased transcription/expression eliminating the factor of increase affected by the cell growth (Figure 2B). GFP expression amounts of the MCP*tac*s promoter clusters in the recombinant strains was also measured via SDS-PAGE (Figure 3). Quantification of the protein band in SDS-PGAE indicated that the expression of GFP was also enhanced with the increase of the number of the core-*tac*-promoter until it reached five, which confirmed the results obtained by the fluorescence/OD_600_. The gradually increased strength of the MCP*tac*s promoter clusters indicated a potential application metabolic engineering.

### Application of the MCP*tac*s promoter cluster in PHB production

The MCP*tac*s promoter cluster was demonstrated its application in metabolic engineering for PHB production in *E. coli*. PHB is known as an intracellular carbon/energy storage compounds and has a huge market potential due to its biodegradability. The metabolic accumulation of PHB from intermediate acetyl-CoA involves three enzymes encoded by the *phaCAB *operon. Generally, high copy number plasmids were used to overexpress the three enzymes to obtain a high production of PHB in recombinant *E.coli*. By expressing the *phaCAB *operon on pBluescript SK, a high-copy plasmid, recombinant *E.coli *can accumulate up to 85.8% PHB of cell dry weight (CDW) [20]. Integration of the *phaCAB *genes into the chromosome increased the stability of the construct but caused dramatically reduced PHB accumulation. Our preliminary experiment showed that single copy integration of PHB operon into the chromosome can only accumulate 3.6% of cell dry weight PHB. Previous study also found that single copy integration of the phbCAB genes in the chromosome caused a very low PHB accumulation [17]. Therefore, we fused the *phaCAB *genes in the downstream of the 5CP*tac*s promoter cluster, which was found to have the strongest transcription strength, and integrated the whole cassette together with the flanking regions (4.3 kb in total size) into the chromosome of *E. coli*, resulting in the strain *E. coli *DH5α/*ΔpoxB*::5TPHB.

**Figure 4:**
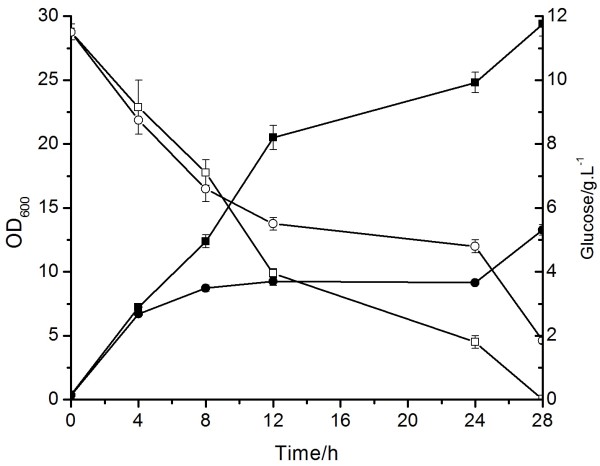
**PHB production in the engineered *E. coli***. Glucose consumption and cell growth*. Solid circles *OD_600 _of DH5α/Δ*poxB*::1TPHB, *solid squares *OD_600 _of DH5α/Δ*poxB*::5TPHB, *open circles *glucose consumption of DH5α/Δ*poxB*::1TPHB, *open squares *glucose consumption of DH5α/Δ*poxB*::5TPHB.

This strain was cultivated in glucose medium and found to accumulate 23.7% PHB (wt% of CDW) after 28 h batch fermentation. The PHB accumulation was 5.6-fold more than that accumulated by the control strain E. coli DH5α/ΔpoxB::1TPHB which integrated single copy core-tac-promoter. Monitoring the fermentation process, we found that consumption of glucose in E. coli DH5α/ΔpoxB::5TPHB was apparently faster than that of the control strain (Figure 4). The faster glucose consummation may contribute to the faster cell growth and high PHB accumulation. In addition, we also analyzed the transcription level of the phaCAB genes integrated into the chromosome of E. coli via RT-PCR. The result showed that the transcription activity of the phaCAB genes in E. coli DH5α/ΔpoxB::5TPHB was around 6-fold increase in compare with the control (Table 1).

**Table 1:** Effect of the 5CP*tac*s promoter on the *phaCAB *genes transcription.

Genes	*phbA*	*phbB*	*phbC*
Relative Expression amount	8.80 ± 0.39	6.62 ± 0.14	7.73 ± 0.15

Expression levels of *phbA, phbB *and *phbC *in DH5α/*poxB*::5TPHB *are *relative to that of the control strain DH5α/*poxB*::1TPHB. The error bars indicate the standard deviation from the mean of the three replicates

## Discussion

In nature, the repetitive sequences including interspersed and tandem repetitive elements usually exist in eukaryotic genomes [21], even in promoter sequence [22]. Tandem repetitive sequences in eukaryotic genomes are involved in various regulation mechanisms of gene transcription and expression [23]. Many tandem repetitive sequences [23] and different promoters arranged in tandem [24,25] are also conserved in prokaryotes. However, there has been no report that tandem repetitive promoter locates in prokaryotes genome by far. In this study, we designed and developed the promoter clusters consisted of the same core-*tac*-promoter arranged in tandem repeats in *E.coli*.

It should be pointed out that the high fidelity Phusion Host Start DNA Polymerase played an important role in assembling the MCP*tac*s promoter clusters with various tandem repeats of the core-*tac*-promoter due to its 3'-5' exonuclease activity. After digesting with restriction enzyme, there were still few unpaired residues at both 3' and 5' ends of the restriction sites at the assemble fragments. Fortunately, except for 5'-3' polymerase activity, Phusion Host Start DNA Polymerase can also remove base sequence, especially the unpaired base, from 3' end of the assemble fragment due to its high fidelity. Meanwhile, T5 exonuclease acted on the 5' end of the assemble fragments to produce partial single strand at the 3' end of the DNA molecular. Thus, the five core*-tac*-promoters may be also sliced one to four. Hence, a random number of the core*-tac*-promoter beginning from one can be constructed (Figure 1).

These promoter clusters exhibited differentially increased transcription strength compared with the original core*-tac-*promoter. Among them, the 5CP*tac*s promoter cluster was found to have strong transcription strength. Further increase in tandem repeats did not improve the transcription strength obviously. This may be due to the promoter occlusion in the process of RNA polymerases recognition and transcription [25,26]. In addition, the inter-promoter space may also be a considerable factor as it is necessary for *E.coli *RNA polymerase to occupy over a region of 80 bp during the initiation of transcription [27]. Thus, the MCPtac promoter clusters may only accommodate limited RNA polymerases.

Due to the weak promoter strength, the existing gene overexpression methods, including plasmids and repeated integration, intended to realize this goal by increasing the copy number of the desired gene [6]. The general plasmid-based overexpression method is not stable and easy-to-lose under the conditions that antibiotics are absent [7,17]. The chemically inducible chromosomal evolution (CIChE) method, aiming to obtain multi-copies of the desired gene in chromosome, was confirmed to be efficient in metabolic engineering and the constructed strains were stable in the absence of antibiotics. However, taking the example of the *phaCAB *genes (about 6.0 kb including the antibiotic gene and the homologous sequence) which was integrated into the chromosome in *E.coli*, the total size of the integrated heterologous DNA reached 120 kb if 20 copies were obtained through evolution, about 2.6% of the size of *E. coli *chromosome. However, this strain can only accumulate about 18% PHB (wt% CDW) [17]. While our engineered strain DH5α/*ΔpoxB*::5TPHB, of which only 4.3 kb heterologous DNA sequence was integrated into the chromosome, can produce 23.7% PHB (wt% CDW). Although they found no growth difference between the engineered strain and the control, the extra synthesis of large amount of DNA should consume abundant nucleotides and much energy [28]. The integrated *phaCAB *genes together with the 5CP*tac*s promoter cluster into the chromosome were also proved to be stable in our strain *E. coli *DH5α, in which *recA *was deleted.

This strategy has many potential applications in the metabolic engineering. We may construct a platform for stable gene overexpression by putting a reverse selection maker under control of the 5CP*tac*s promoter cluster and integrating them into the chromosome of *E. coli*. Then, the desired genes can be used to replace the reverse selection maker through one-step homologous recombination. In addition, two or more different inducible promoters may be fused in tandem; therefore time-dependent expression can be achieved by responding different circumstances or adding corresponding inducers at different times.

## Conclusions

In this study, we designed a strategy which can achieve high-level gene expression by tandem repeating the core*-tac-*promoter. Increasing tandem repeats of the core*-tac*-promoter can enhance the transcription/expression strength of the MCP*tac*s promoter clusters. By integrating of the 5CP*tac*s promoter cluster into the chromosome of *E. coli*, we achieved high and stable expression of the target gene with insertion of a small DNA fragment (only 4.3 kb, taking the *phbCAB *genes for instance). The series of MCP*tac*s promoter clusters can be applied in pathway engineering of *E. coli *and extended to other bacteria.

## Materials and methods

### Bacterial strains, plasmids and oligonucleotides

Bacterial strains and plasmids used in this study were shown in Table 2. All oligonucleotides used in this study were summarized in Table 3.

**Table 2:** Strains and plasmids used in this study

Strains and plasmids	Relevant properties	Source
**Strains** DH5α	F - Φ80 *lacZ*Δ*M15 recA endA1*Δ (*lacZYA-argF*) U169 *deoR gyrA*96 *thi-I hsdR*17 *supE*44 *relAI*	Our laboratory

DH5α/*ΔpoxB*::*tet*	*ΔpoxB*, Tet^R^	This work

DH5α/*ΔpoxB*::1TPHB	*ΔpoxB*::1*tac-phaCAB*	This work

DH5α/*ΔpoxB*::5TPHB	*ΔpoxB*::5*tac-phaCAB*	This work

**Plasmids**		

pGreenTIR	*Plac-*TIR*-gfp *in pUC1813, Ap^R^	[29]

pCL1920	pSC101 replication, Sp^R^	[30]

pLC	pLC1920-derived, ploylinker	This work

pLG	pLC-derived, *gfp *gene from pGreenTIR	This work

p1TG	pLG-derived, 1 single copy of the core*-tac-*promoter	This work

p2TG	pLG-derived, 2 tandem repeats of the core*-tac-*promoter	This work

p3TG	pLG-derived, 3 tandem repeats of the core*-tac-*promoter	This work

p4TG	pLG-derived, 4 tandem repeats of the core*-tac-*promoter	This work

p5TG	pLG-derived, 5 tandem repeats of the core*-tac-*promoter	This work

p6TG	pLG-derived, 6 tandem repeats of the core*-tac-*promoter	This work

p7TG	pLG-derived, 7 tandem repeats of the core*-tac-*promoter	This work

p8TG	pLG-derived, 8 tandem repeats of the core*-tac-*promoter	This work

p9TG	pLG-derived, 9 tandem repeats of the core*-tac-*promoter	This work

p10TG	pLG-derived, 10 tandem repeats of the core*-tac-*promoter	This work

pCP20	FLP^+^, λ *c*I857^+^, λ *p*_R _Rep^ts^; Amp^R^, Cm^R^	[31]

pBHR68	pBluescript II SK^-^-derived, *phaCAB *genes from *Ralstonia eutropha*	[32]

pTKIP	Cloning vector, LP regions, I-SceI restriction sites, Amp^R^, Km^R^	[16]

pTKS/CS	p15A replication, LP regions, I-SceI restriction sites, Cm^R^, Tet^R^	[16]

pTKRED	pSC101 replication, *ParaBAD-*driven I-SceI gene, λ-Red, Sp^R^,	[16]

pTKIP-1TPHB	pTKIP-derived, 1*tac-phbCAB*	This work

**Table 3:** Oligonucleotides used in this study

oligonucleotides	Sequence
*gfp*-F	5'-GCCATGCATAGTAAAGGAGAAGAACTT-3'

*gfp*-R	5'-GCCCTCGAGCTATTTGTATAGTTCATC-3'

P5*tac*-F	5'-CCCGTCTTACTGTCGGGAATTCGTA-3'

P5*tac*-R	5'-TGCATCTAGTATTTCTCCTCTTTAA-3'

*phaCAB*-F	5'-TTAATGCAT GCGACCGGCAAAGGCGCGGCAGCTTCCAC-3'

*phaCAB*-R	5'-ATTCTCGAG TCAGCCCATATGCAGGCCGCCGTTG-3'

*poxB*-F	5'-GCAGGGGGATTTGGTTCTCGCATAATCGCCTTATGCCCGA TGATATTCCTTTCATCGGGCTACGGCCCCAAGGTCCAAAC-3'

*poxB*-R	5'-GCCACCCTTTTTACCTTAGCCAGTTTGTTTTCGCCAGTTCG ATCACTTCATCACCGCGTCTTGGCTTCAGGGATGAGGCG-3'

**RT-PCR**	

*gapA*-F	5'-AACTGAATGGCAAACTGACTGGTA-3'

*gapA*-R	5'-TTTCATTTCGCCTTCAGCAGC-3'

*phbA*-F	5'-CAAGACCTGGACCTGATGGAG-3'

*phbA*-R	5'-GCCGTTCACATTGACCTTGG-3'

*phbB*-F	5'-GTGGTGTTCCGCAAGATGAC-3'

*phbB*-R	5'-CGACGAGATGTTGACGATGC-3'

*phbC*-F	5'-CTGGACTTTGCCGACAC-3'

*phbC*-R	5'-CGTAGTTCCACACCAGG-3'

The 5CP*tac*s promoter cluster's sequence	TTGACAATTAATCATCGGCTCGTATAATGTGTGGAATTGTGTTGACAATTAATCATCGGCTCGTATAATGTGTGGAATTGTGGAGCTCTTGACAATTAATCATCGGCTCGTATAATGTGTGGAATTGTGTTGACAATTAATCATCGGCTCGTATAATGTGTGGAATTGTGTTGACAATTAATCATCGGCTCGTATAATGTGTGGAATTGTG

*Lineate sequence *the core-*tac*-promoter

### Construction of the MCP*tac*s promoter clusters

The construction of the MCP*tac*s promoter clusters was described as follows (Figure 1): The designed ploylinker was synthesized artificially and ligated with two *EcoR*I sites of plasmid pCL1920, resulting the plasmid pLC. The *gfp *gene was amplified from the plasmid pGreenTIR by PCR using the primers *gfp*-F and *gfp*-R and cloned into the *Nsi*I/*Xho*I restricted vector pLC, generating the reporter plasmid pLG. The 5CP*tac*s promoter cluster was synthesized in the form of five core*-tac*-promoters in tandem and inserted into *Hind*III and *BamH*I sites of the plasmid pLG to produce the plasmid p5TG. A one-step method of assembling several overlapping DNA fragments was adopted to construct the different MCP*tac*s promoter clusters [19]. In brief, the DNA fragment with five tandem repeats of the core*-tac*-promoter with flanked extensions was amplified by PCR using the primers P5*tac*-F and P5*tac*-R. Fragment 1 was obtained by digesting the resulting PCR products with *BamH*I, and fragment 2 was generated by cutting with *Hind*III and *BamH*I. The plasmid p5TG was linearized by *Hind*III to produce fragment 3. Then, fragment 1, 2 and 3 were assembled together *in vitro *under the action of T5 exonuclease (Epicentre), Phusion Hot Start DNA Polymerase (New England Biolabs (NEB)) and Taq DNA ligase (NEB) at 50°C for 15 min. The resulting constructs containing different promoters were then transformed into competent cells and were firstly screened based on the fluorescence signal and PCR detection. The finally plasmid were confirmed by double digestion and sequencing.

### Fluorescence assay

Cells harboring the MCP*tac*s promoter clusters were grown in 50 ml Luria broth (1.0% tryptone, 0.5% yeast extract, and 1.0% NaCl) at 220 rpm and 37°C. Samples for measurement were taken out every 2 h and harvested by centrifugation at 14,000 × g for 2 min. After being resuspended with PBS buffer (137 mM NaCl, 2.7 mM KCl, 10 mM Na_2_HPO_4_, 2 mM KH_2_PO_4_, pH 7.4), 200 μl of bacterial culture was transferred into a 96-well plate in which OD_600 _and fluorescence were read with excitation at 485 nm and emission at 528 nm using a Multi-Detection Microplate Reader, Synergy HT (BioTek). For each sample, 3 repetitions were performed with PBS as a blank. Average of three repeats for the specific fluorescence and OD_600 _were chosen as the reference of promoter strength.

### Integration of the *phaCAB *genes into chromosome

Integration of the *phaCAB *genes was executed by following Kuhlman's method [16]. In brief, the *poxB *gene of DH5α was replaced by a tetracycline resistance gene (*tet*) flanked by I-SceI recognition site and 25 bp of landing pad region at each side, amplified from the plasmid pTKS/CS, and *E. coli *DH5α(*ΔpoxB*::tet) was obtained. The *phaCAB *genes cloned from pBHR68 was inserted into *Nsi*I and *Xho*I sites of p1TG, resulting plasmid p1TPHB. Both the plasmid pTKIP and p1TPHB were digested with *Hind*III and *EcoR*I and ligated together to create the plasmid pTKIP-1TPHB. Then, the plasmid pTKIP-1TPHB was transformed in DH5α (*ΔpoxB*::tet)/pTKRED. *In vivo *recombination between landing pad regions was finished under the action of RED recombinase and the stimulation effect of I-SceI, resulting the strain DH5α/*ΔpoxB*::1TPHB-*kan*. The kanamycin resistance gene was eliminated with the help of pCP20 cultivating at 42°C as described by Datshenko [33] and the finally strain DH5α/*ΔpoxB*::1TPHB was obtained. In the same way, the strain DH5α/*ΔpoxB*::5TPHB was constructed.

### PHB fermentation

Preculture was grown overnight at 37°C, 250 rpm in Luria broth medium supplemented with 25 μg/ml kanamycin. Fermentation was carried out in 250 ml shake flask containing 50 ml modified M9 medium (42.3 mM Na_2_HPO_4_·12H_2_O, 22.0 mM KH_2_PO_4_, 8.6 mM NaCl, 18.7 mM NH_4_Cl, 2% extract, 1 mM MgSO_4_, 0.1 mM CaCl_2_) supplemented with 1.2% glucose, 0.1 M MOPS and 25 μg/ml kanamycin antibiotic at 37°C and 250 rpm. pH was adjusted at 7.0 with NH_3_·H_2_O. Samples with a defined interval were harvested by centrifugation at 14,000 × g for 2 min. The supernatant was then diluted to an appropriate concentration to measure glucose concentration using SBA-40 C (Biology Institute of Shandong Academy of Sciences). After suspending with PBS (pH 7.4), the sample cells were read at 600 nm for the optical density (OD_600_).

### Analysis of PHB by GC

The PHB content was quantitatively determined via gas chromatography (GC, Shimadzu) using a Gas Chromatography. Cultures were collected by centrifugation (5,000 × g, 10 min) and lyophilized overnight. Mixture of 20 mg lyophilized cells mass with 1 ml chloroform, 1 ml methanol and 15% (v/v) sulfuric acid was boiled for 1 h for methylation. Then 1 ml ddH_2_O was added to the mixture and shaken sharply for 20 s. After the standing and layering process, the organic phase was taken out and was analyzed by GC [34].

### QRT-PCR assay of the *phaCAB *genes

The *phaCAB *genes were assessed in transcription level via Quantitative Reverse Transcription PCR (QRT-PCR). Cells for mRNA preparation were cultivated for 4 h and then harvested. Total mRNA of DH5α/Δ*poxB*::1TPHB and DH5α/Δ*poxB*::5TPHB were extracted using the RNeasy Mini Kit (Tiangen). The cDNA was amplified through reverse transcription with the total mRNA as the templates. Quantity real-time PCR (QPCR) amplification primers were designed and were listed in Table 3. The *gapA *gene was chosen as the control for normalization. QPCR was performed in a 96-well plate with a reaction volume of 20 μl for each sample in MyiQ5768R Real-Time PCR detection system using a SYBRs Premix Ex Taq TM II (Perfect Real Time), according to manufacturer's specification (TaKaRa). The obtained data were analyzed by using the 2^-ΔΔCt ^method described previously [35].

## Abbreviations

MCP*tac*s: multiple core-*tac*-promoters; 5CP*tac*s: five core-*tac*-promoters

## Competing interests

The authors declare that they have no competing interests.

## Authors' contributions

ML carried out most of the experiments and wrote the manuscript. JW and YG are responsible for performing some experiments. YL participated in the design of this study. QW and QL assisted in data analysis and revised the manuscript. QQ conceived of the study, participated in its design, coordination and helped to draft the manuscript. All authors read and approved the final manuscript.
